# Supplementary material for: Causal Relationship between Aging and Anorexia Nervosa: A White-Matter-Microstructure-Mediated Mendelian Randomization Analysis
Source: Biomedicines. 2024 Aug 16;12(8):1874. doi: 10.3390/biomedicines12081874 (PMC11351342; doi:10.3390/biomedicines12081874)
Supplement: Supplementary file 1 [file biomedicines-12-01874-s001.zip › Supplementary Figure/Figure S4/Singe SNP plot of MR analysis for TL to ubm-b-1728.pdf]

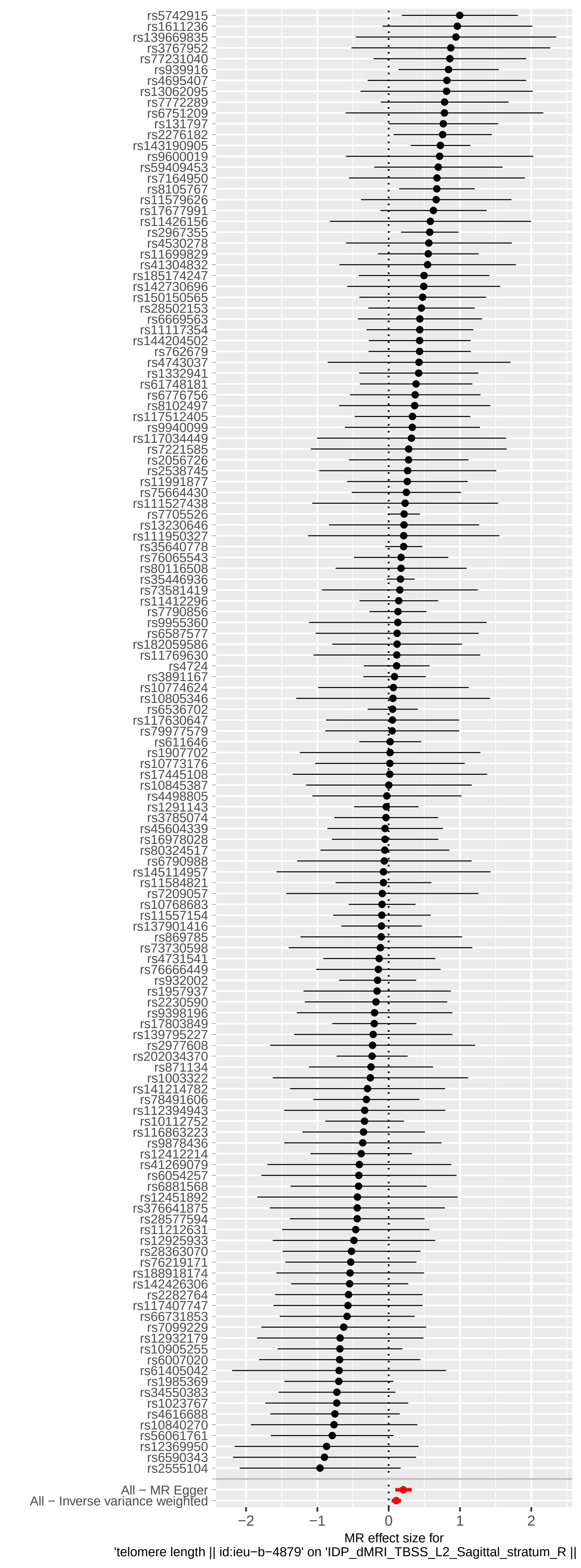
rs5742915
rs1611236
rs139669835
rs3767952
rs77231040
rs939916
rs4695407
rs13062095
rs7772289
rs6751209
rs131797
rs2276182
rs143190905
rs9600019
rs59409453
rs7164950
rs8105767
rs11579626
rs17677991
rs11426156
rs2967355
rs4530278
rs11699829
rs41304832
rs185174247
rs142730696
rs150150565
rs28502153
rs6669563
rs11117354
rs144204502
rs762679
rs4743037
rs1332941
rs61748181
rs6776756
rs8102497
rs117512405
rs9940099
rs117034449
rs7221585
rs2056726
rs2538745
rs11991877
rs75664430
rs111527438
rs7705526
rs13230646
rs111950327
rs35640778
rs76065543
rs80116508
rs35446936
rs73581419
rs11412296
rs7790856
rs9955360
rs6587577
rs182059586
rs11769630
rs4724
rs3891167
rs10774624
rs10805346
rs6536702
rs117630647
rs79977579
rs611646
rs1907702
rs10773176
rs17445108
rs10845387
rs4498805
rs1291143
rs3785074
rs45604339
rs16978028
rs80324517
rs6790988
rs145114957
rs11584821
rs7209057
rs10768683
rs11557154
rs137901416
rs869785
rs73730598
rs4731541
rs76666449
rs932002
rs1957937
rs2230590
rs9398196
rs17803849
rs139795227
rs2977608
rs202034370
rs871134
rs1003322
rs141214782
rs78491606
rs112394943
rs10112752
rs116863223
rs9878436
rs12412214
rs41269079
rs6054257
rs6881568
rs12451892
rs376641875
rs28577594
rs11212631
rs12925933
rs28363070
rs76219171
rs188918174
rs142426306
rs2282764
rs117407747
rs66731853
rs7099229
rs12932179
rs10905255
rs6007020
rs61405042
rs1985369
rs34550383
rs1023767
rs4616688
rs10840270
rs56061761
rs12369950
rs6590343
rs2555104
All − MR Egger
All − Inverse variance weighted
−2
−1
0
1
2
MR effect size for
'telomere length || id:ieu−b−4879' on 'IDP_dMRI_TBSS_L2_Sagittal_stratum_R ||
